# Supplementary material for: Metabolic control of progenitor cell propagation during Drosophila tracheal remodeling
Source: Nat Commun. 2022 May 20;13:2817. doi: 10.1038/s41467-022-30492-4 (PMC9122933; doi:10.1038/s41467-022-30492-4)
Supplement: Supplementary file 1 — Supplementary Information [file 41467_2022_30492_MOESM1_ESM.pdf]

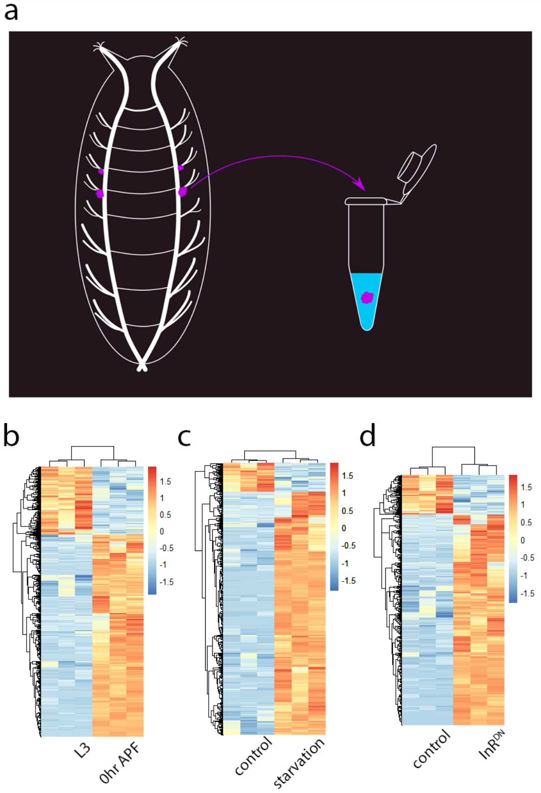
a
b
c
d
1.5
1
0.5
0
-0.5
-1
-1.5
L3
0hr APF
control
starvation
control
InR^DN

Supplementary Figure 1. RNA-seq of a cluster of tracheal progenitors. a, Schematic diagram illustrating the procedure of isolating ~10 Tr5 progenitors (magenta) and RNA preparation. **b**-**d**, RNA-seq identified differentially expressed genes by pupariation (**b**), starvation (**c**), and expression of InR^{DN} (**d**).

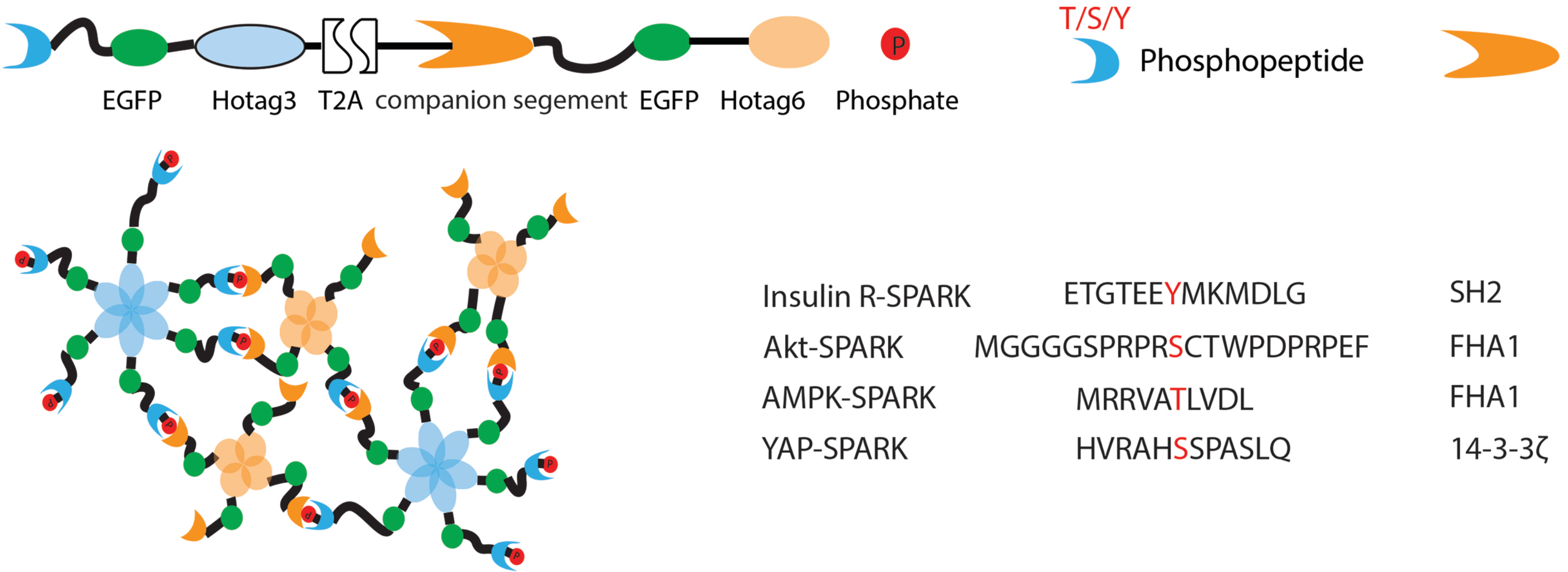

EGFP
Hotag3
T2A
companion segement
EGFP
Hotag6
P
Phosphate
T/S/Y
Phosphopeptide
Insulin R-SPARK ETGTEEYMKMDLG SH2
Akt-SPARK MGGGGSPRPRSCTWPDPRPEF FHA1
AMPK-SPARK MRRVATLVDL FHA1
YAP-SPARK HVRAHSSPASLQ 14-3-3ζ

Supplementary Figure 2. Rational design of various SPARK sensor. EGFP fluorophore and homo-oligomeric tag (Hotag)3 are tandem conjugated to a phosphopeptide-containing substrate sequence. A phosphopeptide-binding domain is fused to Hotag6. These two segments were interconnected by a self-cleaving 2A sequence. The consensus sequence of substrate domain and phosphorylation recognition domain are adopted from following references: insulin receptor [56], Akt [57], and AMPK [58].

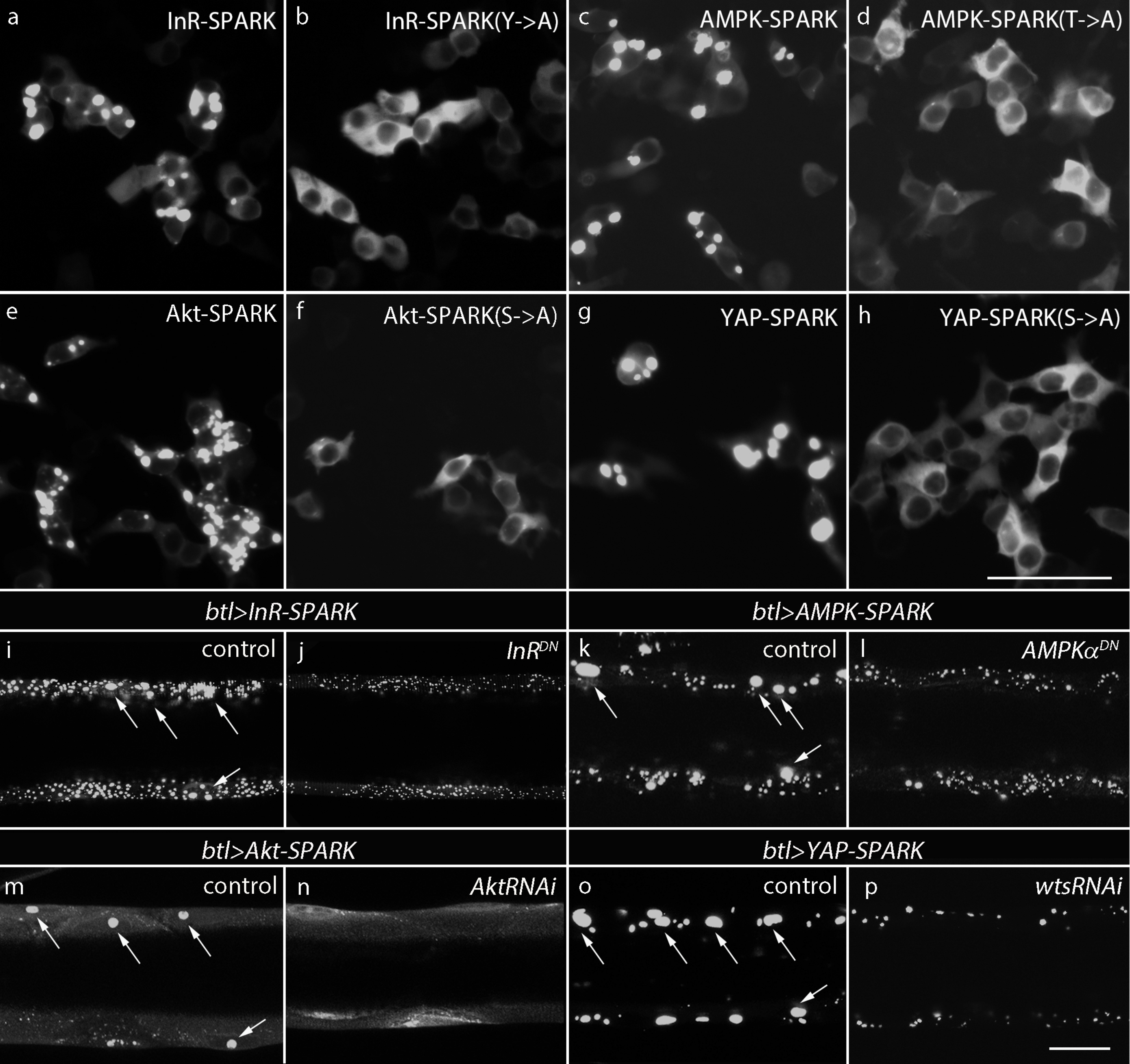

a
InR-SPARK
b
InR-SPARK(Y->A)
c
AMPK-SPARK
d
AMPK-SPARK(T->A)
e
Akt-SPARK
f
Akt-SPARK(S->A)
g
YAP-SPARK
h
YAP-SPARK(S->A)
btl>InR-SPARK
btl>AMPK-SPARK
i
control
j
InR^DN
k
control
l
AMPKα^DN
btl>Akt-SPARK
btl>YAP-SPARK
m
control
n
AktRNAi
o
control
p
wtsRNAi

Supplementary Figure 3. Validation of various SPARK sensors in HEK293T cells and *Drosophila*. a-h, Representative confocal images showing the GFP droplets of SPARK sensors in HEK293T cells. The amount of GFP is unchanged. Diffuse GFP and droplets indicate phosphorylation status of substrate. InR-SPARK (**a**), AMPK-SPARK (**c**), Akt-SPARK (**e**), and YAP-SPARK (**g**). **b**,**d**,**f**,**h**, The GFP droplets were absent when phosphotyrosine/phosphothreonine/phosphoserine was mutated to alanine. **i-p**, Visualization of various SPARK sensors in *Drosophila* trachea. Expression of InR-SPARK (**i**,**j**), AMPK-SPARK (**k**,**l**), Akt-SPARK (**m**,**n**), and YAP-SPARK (**o**,**p**) in *Drosophila* trachea. Signals of SPARK reporters are reduced upon the perturbation of upstream kinases. Arrows denote the GFP droplets of SPARK sensors in *Drosophila* trachea. Scale bars: 50 μm (**a-h**), 20 μm (**i-p**). **a-p**, Three independent experiments were repeated with similar results. Genotype: (**i**) *btl-Gal4/+; tub-Gal80^{ts}/UAS-InR-SPARK*; (**j**) *btl-Gal4/UAS-InR^{DN}; tub-Gal80^{ts}/UAS-InR-SPARK*; (**k**) *btl-Gal4/+; UAS-AMPK-SPARK/+*; (**l**) *btl-Gal4/+; UAS-AMPK-SPARK/UAS-AMPKα^{DN}*; (**m**) *btl-Gal4/tub-Gal80^{ts}; UAS-Akt-SPARK/+*; (**n**) *btl-Gal4/tub-Gal80^{ts}; UAS-Akt-SPARK/UAS-AktRNAi*; (**o**) *btl-Gal4/+; UAS-YAP-SPARK/+*; (**p**) *btl-Gal4/ UAS-wtsRNAi; UAS-YAP-SPARK/+*.

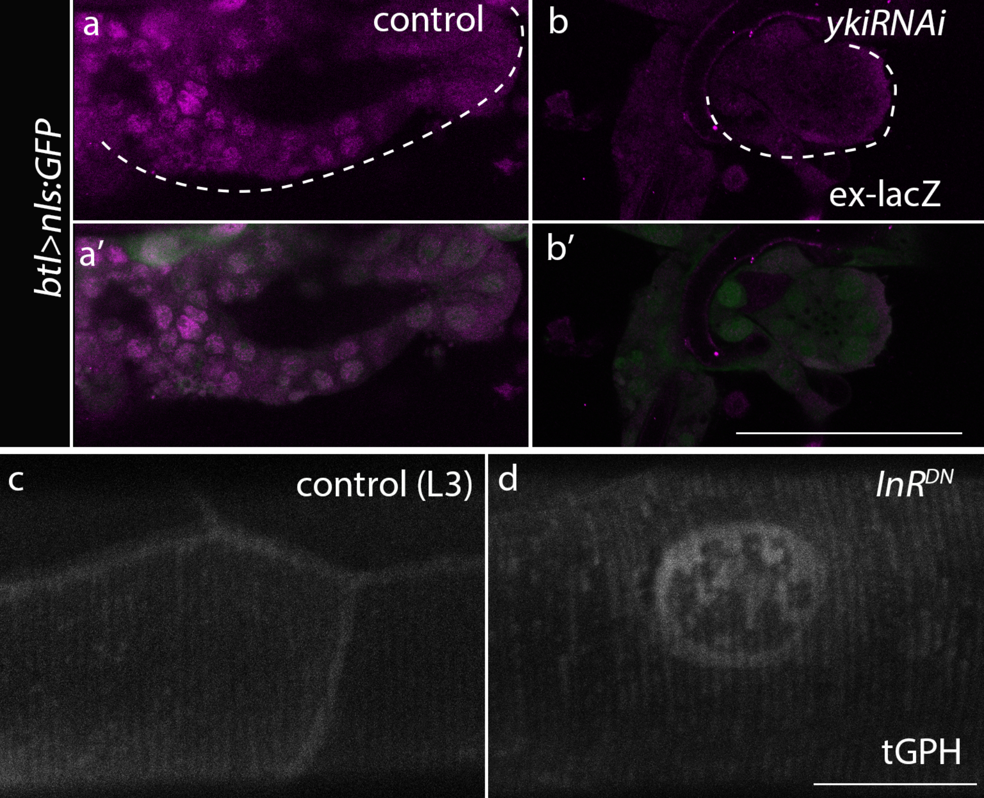

btl>nls:GFP
a
control
a'
b
ykiRNAi
ex-lacZ
b'
c
control (L3)
d
InR^DN
tGPH

Supplementary Figure 4. Perturbation of Yki or insulin signaling in the trachea. **a**-**b’**, The expression of ex-lacZ is decreased in the *ykiRNAi* flies. Staining the trachea of control (**a**,**a**’) and *ykiRNAi* (**b**,**b’**) pupae with antibodies against β-Galactosidase. **a’**,**b’**, Merge image. The progenitors are outlined by dashed lines. **c**,**d**, The membrane localization of tGPH in control larvae and nuclear localization of tGPH in InR^{DN}-expressing larvae. Confocal images showing the dorsal trunk of larval trachea. **a**-**d**, Three independent experiments were repeated with similar results. Scale bars: 50 μm (**a**-**b’**), 20 μm (**c**,**d**).

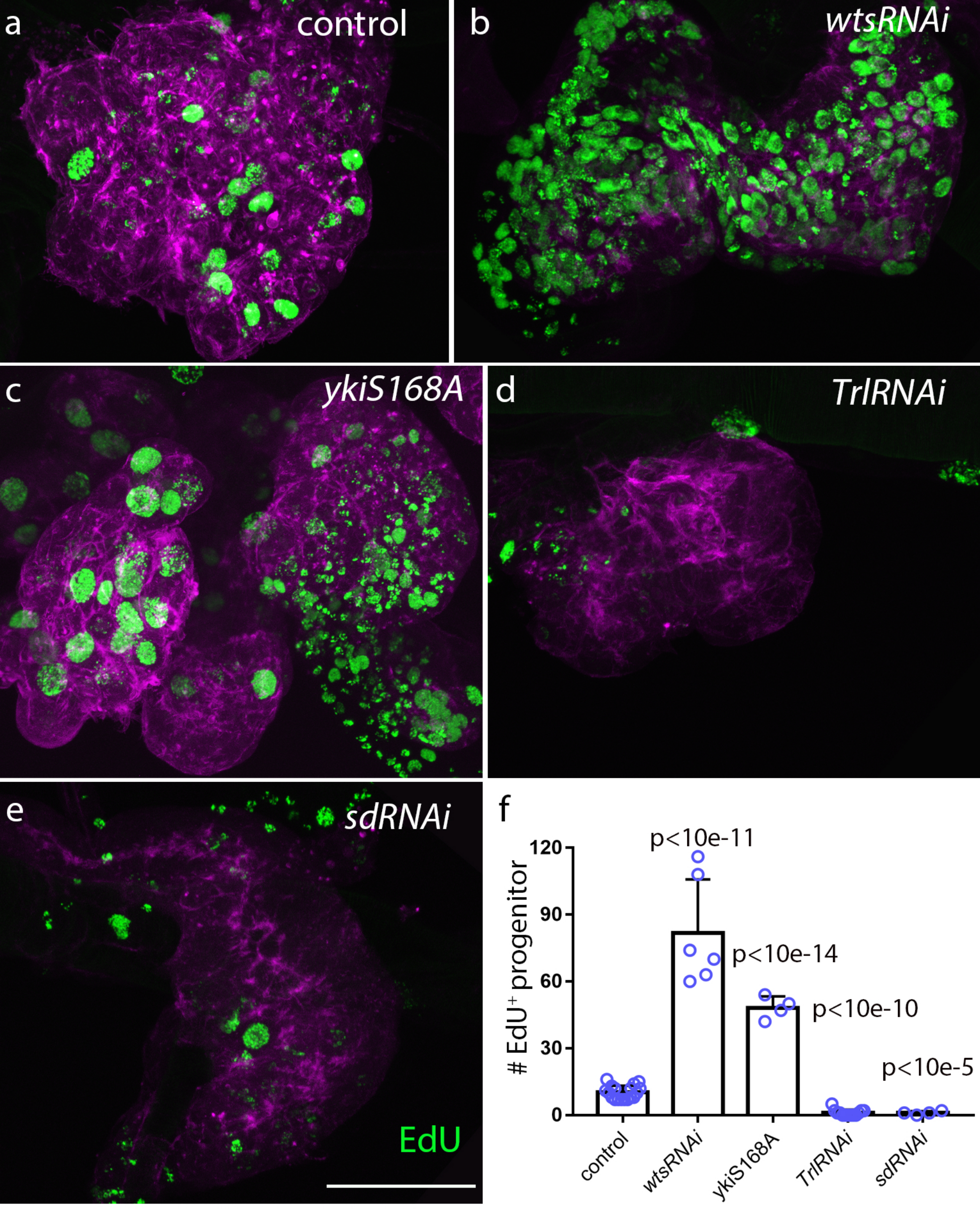

a
control
b
wtsRNAi
c
ykiS168A
d
TrlRNAi
e
sdRNAi
EdU
f
EdU+ progenitor
120
90
60
30
0
p<10e-11
p<10e-14
p<10e-10
p<10e-5
control
wtsRNAi
ykiS168A
TrlRNAi
sdRNAi

Supplementary Figure 5. Dependence of tracheal progenitors on Yki signaling. The proliferation of tracheal progenitors is affected upon the perturbation of Yki signaling. The Yki signaling was activated by interfering against *wts*, expressing the constitutive active form of Yki, or was decreased by depletion of Sd. **a**-**e**, Staining tracheal progenitors of control (**a**), *wtsRNAi* (**b**), *ykiS168A* (**c**), *sdRNAi* (**d**) and *TrlRNAi* (**e**) with antibodies against EdU. **f**, Bar graph plots the number of EdU incorporation in progenitors in contrl (n = 18), *wtsRNAi* (n = 6; *p* = 9.46e-12), *ykiS168A* (n = 4; *p* = 8.89e-15), *TrlRNAi* (n = 13; *p* = 5.45e-11) and *sdRNAi* (n = 4; *p* = 8.04e-6). Three biologically independent experiments were performed. Data are presented as mean values ± SD. Unpaired two-tailed *t*-test was used for all statistical analyses. No adjustments were made for multiple comparisons. Scale bars: 30 μm. Genotypes: (**a**) *btl-Gal4/+; P[B123]-RFP-moe/+*; (**b**) *btl-Gal4/UAS-wtsRNAi; P[B123]-RFP-moe/+*; (**c**) *btl-Gal4/+; P[B123]-RFP-moe/UAS-ykiS168A*; (**d**) *btl-Gal4/UAS-TrlRNAi; P[B123]-RFP-moe/+*; (**e**) *btl-Gal4/+; P[B123]-RFP-moe/UAS-sdRNAi*. Source data for (**f**) are provided as a Source Data file.

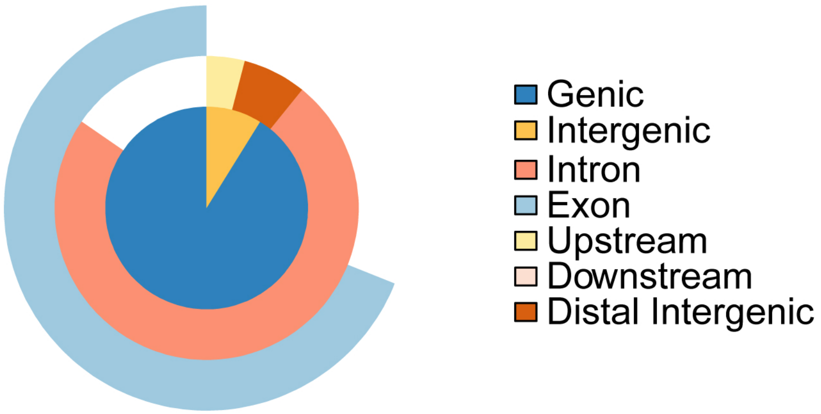
Genic
Intergenic
Intron
Exon
Upstream
Downstream
Distal Intergenic

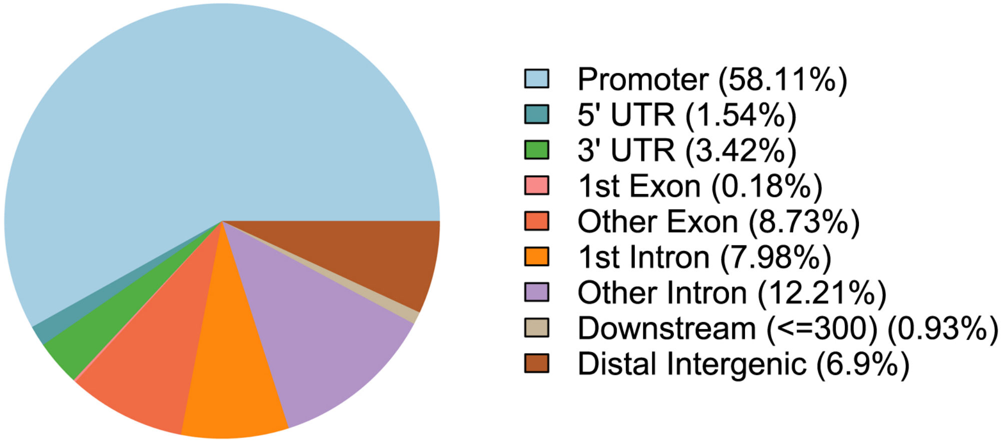
Promoter (58.11%)
5' UTR (1.54%)
3' UTR (3.42%)
1st Exon (0.18%)
Other Exon (8.73%)
1st Intron (7.98%)
Other Intron (12.21%)
Downstream (<=300) (0.93%)
Distal Intergenic (6.9%)

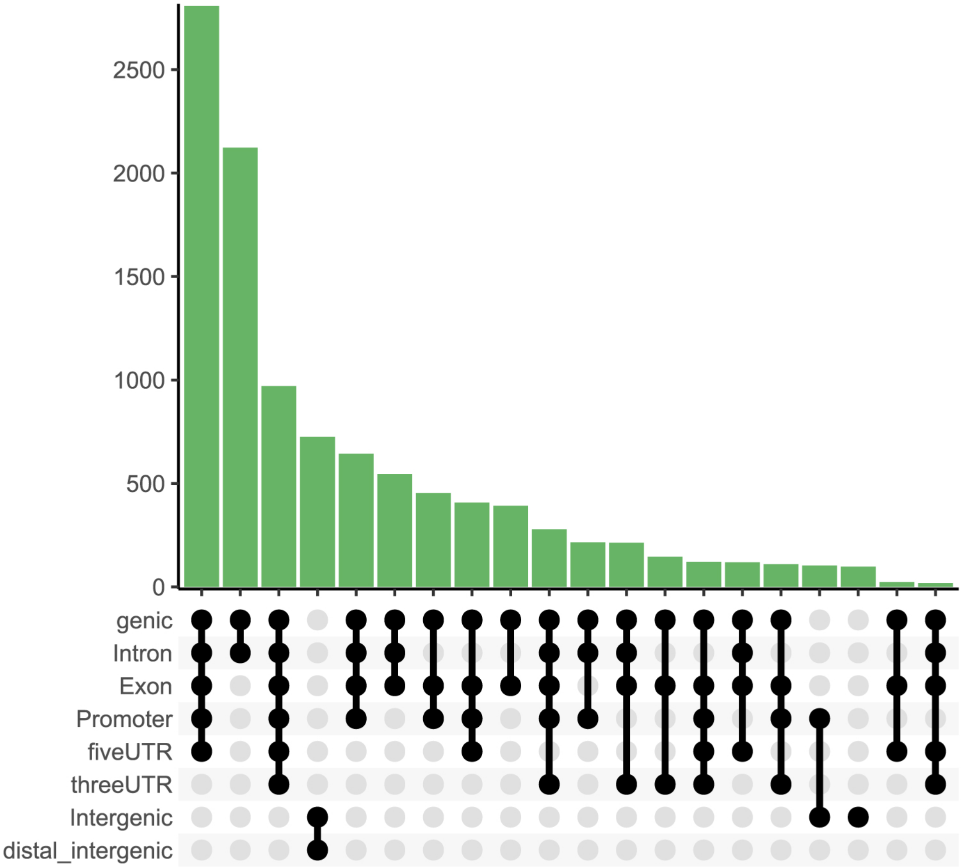
2500
2000
1500
1000
500
0
genic
Intron
Exon
Promoter
fiveUTR
threeUTR
Intergenic
distal_intergenic

Supplementary Figure 6. Annotation and analysis of genomic occupancy of Yki.

Pie chart (upper panel) and histogram (bottom panel) depicting the distribution of ChIP-seq peaks relative to the nearby annotated gene.

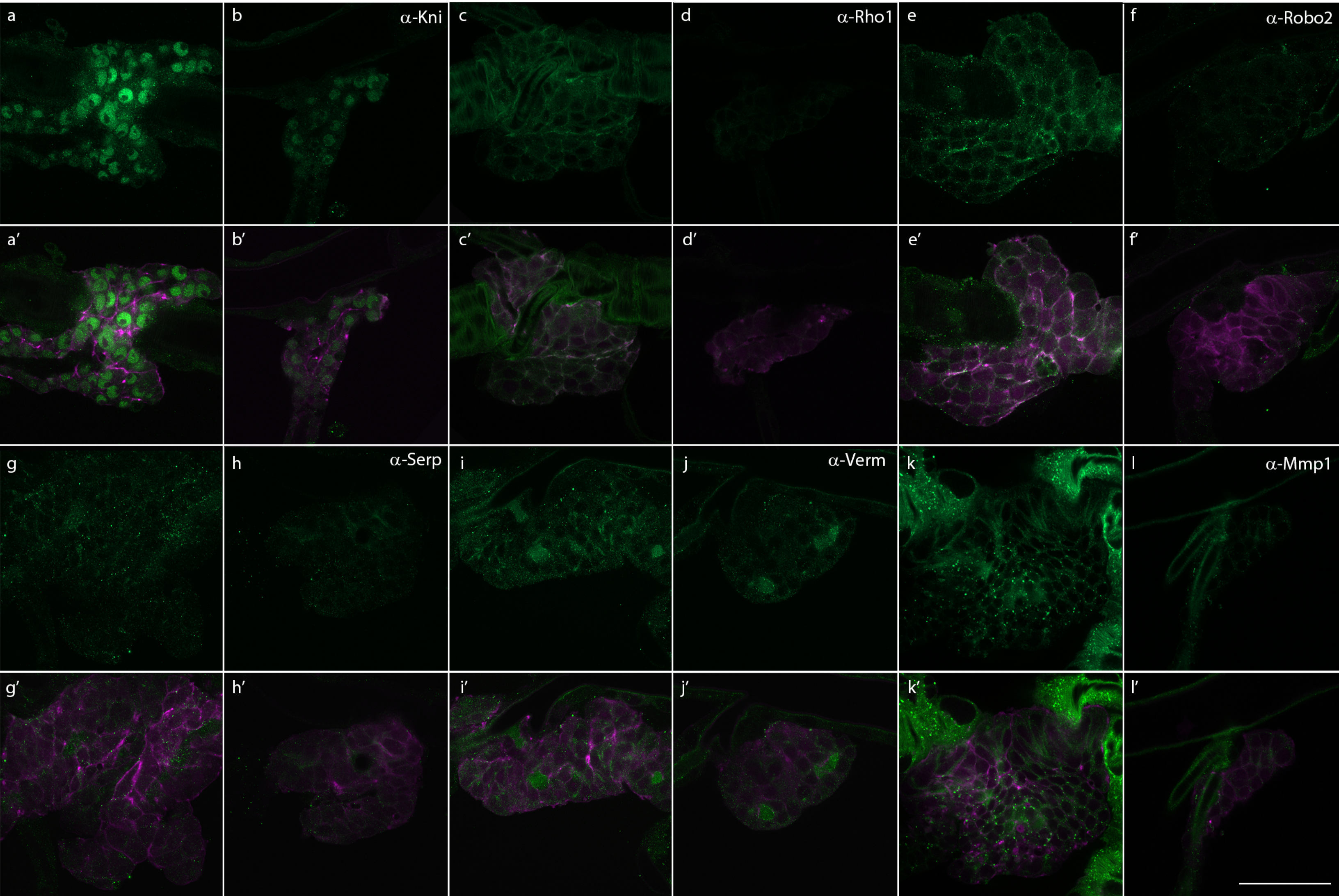

a
b
α-Kni
c
d
α-Rho1
e
f
α-Robo2
a′
b′
c′
d′
e′
f′
g
h
α-Serp
i
j
α-Verm
k
l
α-Mmp1
g′
h′
i′
j′
k′
l′

Supplementary Figure 7. Diverse targets of Yki in tracheal progenitors. Staining of tracheal progenitors with α-Kni (**a**-**b'**), α-Rho1 (**c**-**d'**), α-Robo2 (**e**-**f'**), α-Serp (**g**-**h'**), α-Verm (**i**-**j'**), and α-MMP1 (**k**-**l'**). **a**-**l**, Three independent experiments were repeated with similar results. Scale bar: 50 μm. Genotypes:
(**a**,**a'**,**c**,**c'**,**e**,**e'**,**g**,**g'**,**i**,**i'**,**k**,**k'**) *btl-Gal4/+; P[B123]-RFP-moe/tub-Gal80*^{ts};
(**b**,**b'**,**d**,**d'**,**f**,**f'**,**h**,**h'**,**j**,**j'**,**l**,**l'**) *btl-Gal4/UAS-ykiRNAi; P[B123]-RFP-moe/tub-Gal80*^{ts}.

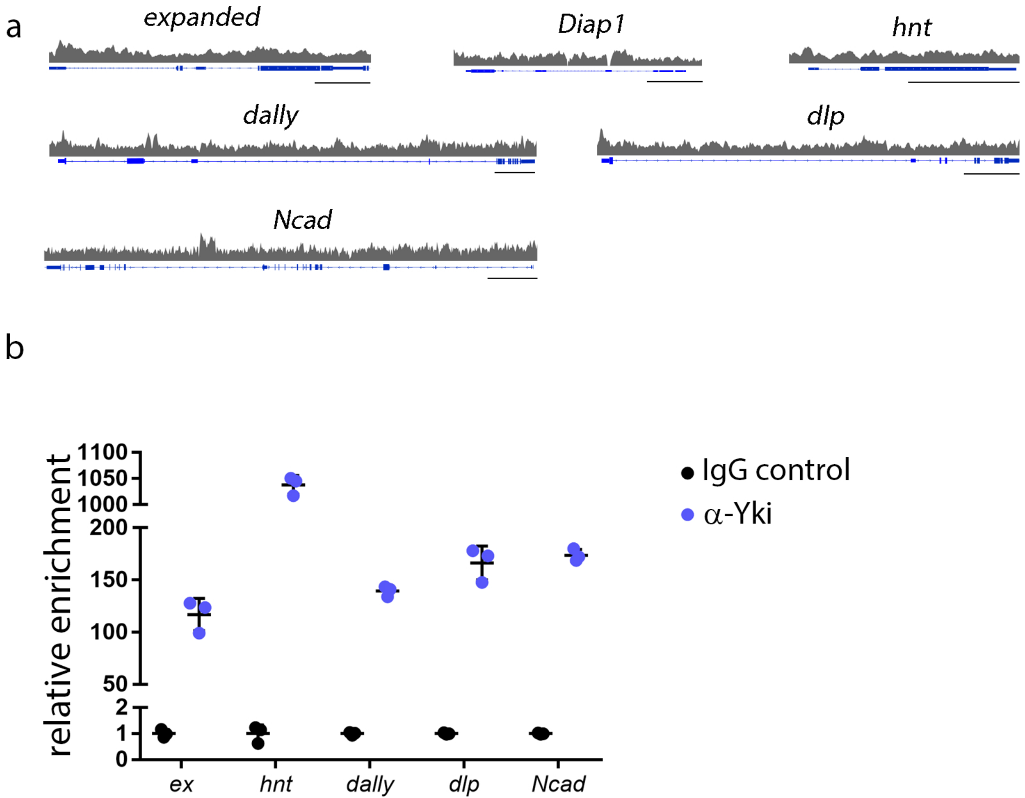
a
expanded
Diap1
hnt
dally
dlp
Ncad
b
relative enrichment
1100
1050
1000
200
150
100
50
2
1
0
ex
hnt
dally
dlp
Ncad
IgG control
α-Yki

Supplementary Figure 8. Association of Yki on binding regions. a, Analysis of genomic input for ChIP-seq. Scale bar: 5 kb, except for *Ncad* (10 kb). **b**, Relative enrichment of Yki on binding regions of target genes compared with IgG control were analyzed by ChIP-qPCR. Three biologically independent experiments were performed. n = 500. Data are presented as mean values ± SD. *p* value: *ex* (*p* = 9.76e-6), *hnt* (*p* = 5.88e-8), *dally* (*p* = 1.05e-6), *dlp* (*p* = 6.07e-5) and *Ncad* (*p* = 8.04e-7). Unpaired two-tailed *t*-test was used for all statistical analyses. No adjustments were made for multiple comparisons. Source data for (**b**) are provided as a Source Data file.

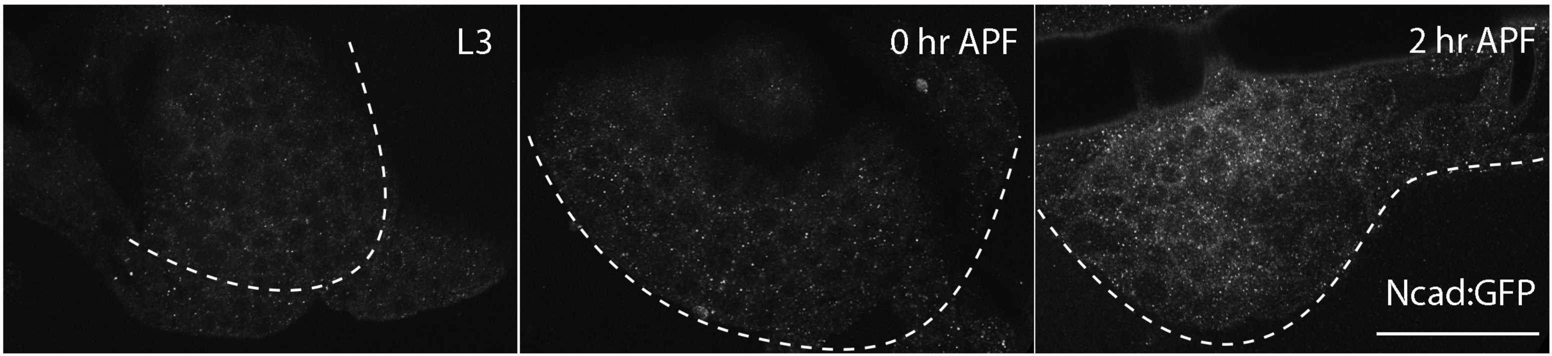
L3
0 hr APF
2 hr APF
Ncad:GFP

Supplementary Figure 9. Fluorescent images of tracheal progenitors expressing Ncad:GFP under the control of endogenous promoter. The progenitors are outlined by dashed lines. Three independent experiments were repeated with similar results in this figure. Scale bars: 30 μm. Genotype: *Ncad:GFP/CyO*.

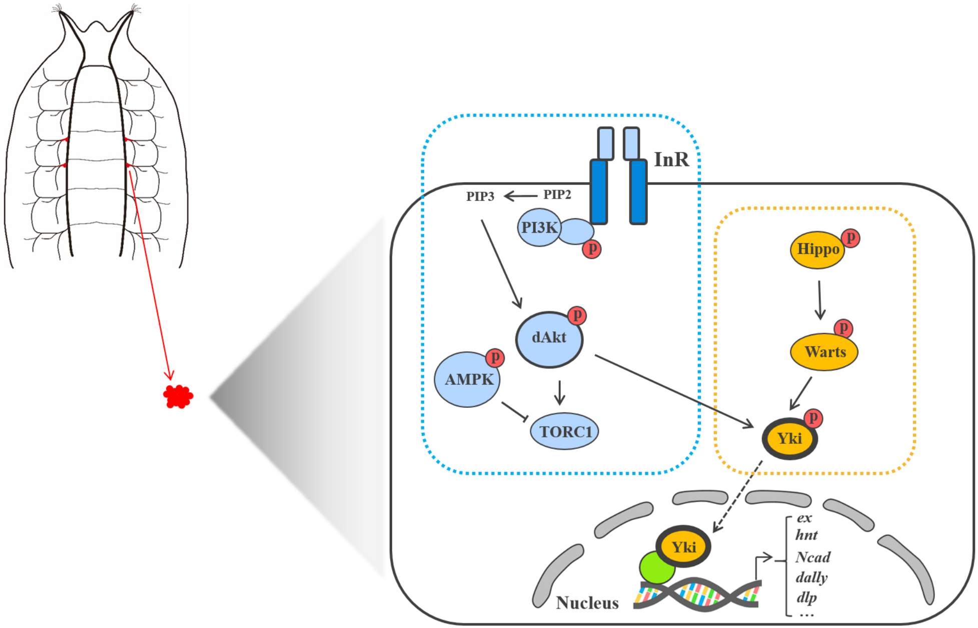
InR
PIP3
PIP2
PI3K
p
dAkt
AMPK
TORC1
Hippo
Warts
Yki
Nucleus
ex
hnt
Ncad
dally
dlp
...

Supplementary Figure 10. Schematic representation depicts the intersection between insulin pathway and Yki signaling to activate tracheal progenitors. Akt-dependent phosphorylation of Yki negatively regulates the activity of Yki and the transcription of its target genes. Blue box comprises key components of insulin pathway. Major components of Hippo pathway are boxed in yellow dashed lines.

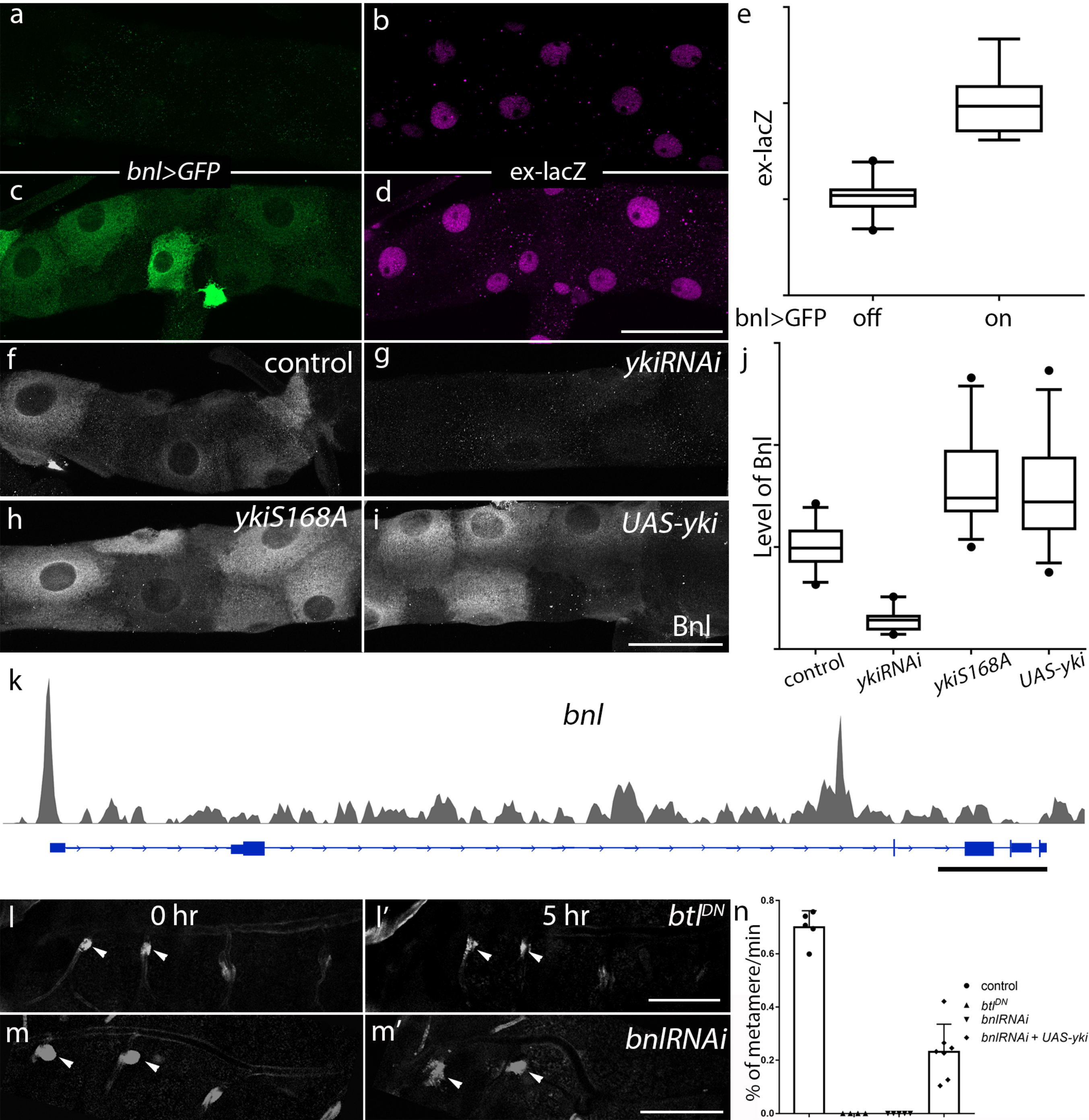

a
b
e
bnl>GFP
ex-lacZ
c
d
ex-lacZ
bnl>GFP off on
f
control
g
ykiRNAi
j
h
ykiS168A
i
UAS-yki
Bnl
Level of Bnl
control
ykiRNAi
ykiS168A
UAS-yki
k
bnl
l
0 hr
l′
5 hr
btl^DN
n
m
m′
bnlRNAi
% of metamere/min
0.8
0.6
0.4
0.2
0.0
control
btl^DN
bnlRNAi
bnlRNAi + UAS-yki

Supplementary Figure 11. Interplay between FGF and Yki signaling in the trachea. a-d, Fluorescent images show the expression of ex-lacZ in Bnl-expressing branch (**c**,**d**) or branch in the absence of bnl expression (**a**,**b**). **e**, Box plots showing the expression of ex-lacZ in trachea branches with (n = 15) or without *bnl>GFP* (n = 22). Four biologically independent experiments were performed. Results are presented as median with minima and maxima. 25th ~ 75th percentile (box) and 5th ~ 95th percentile (whiskers) as well as outliers are indicated in the box plots. p = 3.98e-14. **f-j**, The level of Bnl is affected by Yki signaling. **f-i**, Confocal images showing abundance of Bnl in Tr6 metamere (7hr APF). The level of *bnl* expression was visualized by Bnl immunostaining. The expression of *bnl* in control (**f**), *ykiRNAi* (**g**), *ykiS168A* (**h**) and *UAS-yki* (**i**) pupae. **j**, Box plots represent the relative level of bnl expression in control (n = 29), *ykiRNAi* (n = 22; p = 6.34e-20), *ykiS168A* (n =27; p = 3.13e-9) and *UAS-yki* (n = 31; p = 5.21e-7). Five biologically independent experiments were performed. Data are presented as median with minima and maxima. 25th ~ 75th percentile (box) and 5th ~ 95th percentile (whiskers) as well as outliers are indicated in the box plots. **k**, ChIP-seq peaks illustrating the localization of Yki in the promoter region of *bnl*. Scale bar: 5 kb. **l-m**, Fluorescent images showing the localization of tracheal progenitors in a btl^{DN} (**l**,**l'**) or *bnlRNAi* (**m**,**m'**) fly at 0hr APF (**l**,**m**) and 5hr APF (**l**',**m'**). Arrowheads point to tracheal progenitors. Scale bars: 50 μm (**a-d**, **f-i**), 300 μm (**l-m'**). **n**, Box graph plots the velocity of migrating progenitors in control (n = 5), btl^{DN} (n = 4; p = 9.25e-8), *bnlRNAi* (n = 5; p = 6.43e-9) and *bnlRNAi+UAS-yki* flies (n = 7; p = 4.38e-6). Three biologically independent experiments were performed. Data are presented as mean values ± SD. **e**,**j**,**n**, Unpaired two-tailed *t*-test was used for all statistical analyses. No adjustments were made for multiple comparisons. Source data for (**e**,**j**,**n**) are provided as a Source Data file.

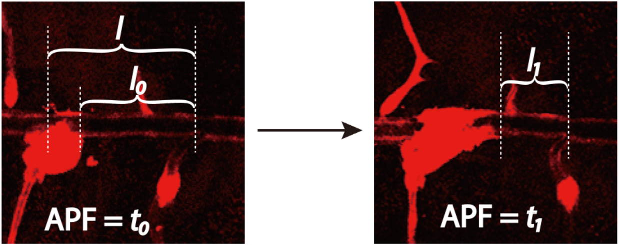

$$v = \Delta l / (l \Delta t)$$

v # migration velocity (% of metamere min^{-1})

$\Delta l = l_0 - l_1$ # distance of migration

l # length of a metamere

$\Delta l / l$ # % of metamere

$\Delta t = t_1 - t_0$ # time of migration (min)

Supplementary Figure 12. Schematic diagram depicts the measurement of migration velocity of tracheal progenitors.
