# Supplementary material for: Metabolic control of progenitor cell propagation during Drosophila tracheal remodeling
Source: Nat Commun. 2022 May 20;13:2817. doi: 10.1038/s41467-022-30492-4 (PMC9122933; doi:10.1038/s41467-022-30492-4)
Supplement: Supplementary file 3 — Description of Additional Supplementary Files [file 41467_2022_30492_MOESM3_ESM.pdf]

Description of Additional Supplementary Files

File Name: Supplementary Movie 1
Description: **The migration of tracheal progenitors (red) on dorsal trunk (DT, Green).** Time-lapse imaging of a white pupa. Progenitors that were situated at Tr4 and Tr5 metameres at 0hr APF were labeled by RFP-moesin and were imaged through the cuticle. Confocal images were captured every 10 minutes for 5 hours. Genotype: *UAS-CD8:GFP; Dl-Gal4, P[B123]-RFP-moe*. Scale bars: 100 µm.

File Name: Supplementary Movie 2
Description: **Fucci fly showing the tracheoblasts that are in mitotic cycle.** 5-hr Time-lapse imaging of mitotic tracheoblasts (green). Genotype: *btl-Gal4/+; P[B123]-RFP-moe/UAS-S/G2/M-Green*. Scale bars: 100 µm.

File Name: Supplementary Movie 3
Description: **The movement of tracheal progenitors in control, *ykiRNAi*, *dallyRNAi*, *dlpRNAi*, *NcadRNAi*, or *sdRNAi* flies.** The migration of tracheal progenitors was impaired by knockdown of *yki*, *dally*, *dlp*, or *Ncad*. Tracheal progenitors do not migrate in the presence of *sdRNAi*. Scale bars: 300 µm.

File Name: Supplementary Movie 4
Description: **The defective movement of tracheal progenitors in Btl^{DN}-expressing flies.** The migration of tracheal progenitors was abolished upon the expression of *btl^{DN}*. Scale bars: 300 µm.
