# Supplementary material for: Metabolic control of progenitor cell propagation during Drosophila tracheal remodeling
Source: Nat Commun. 2022 May 20;13:2817. doi: 10.1038/s41467-022-30492-4 (PMC9122933; doi:10.1038/s41467-022-30492-4)
Supplement: Supplementary file 8 — Reporting Summary [file 41467_2022_30492_MOESM8_ESM.pdf]

nature portfolio

Corresponding author(s): Hai Huang, Qiang Zhang

Last updated by author(s): Apr 23, 2022

Reporting Summary

Nature Portfolio wishes to improve the reproducibility of the work that we publish. This form provides structure for consistency and transparency in reporting. For further information on Nature Portfolio policies, see our Editorial Policies and the Editorial Policy Checklist.

Statistics

For all statistical analyses, confirm that the following items are present in the figure legend, table legend, main text, or Methods section.

n/a	Confirmed	
☐	☒	The exact sample size (*n*) for each experimental group/condition, given as a discrete number and unit of measurement
☐	☒	A statement on whether measurements were taken from distinct samples or whether the same sample was measured repeatedly
☐	☒	The statistical test(s) used AND whether they are one- or two-sided<br>*Only common tests should be described solely by name; describe more complex techniques in the Methods section.*
☒	☐	A description of all covariates tested
☐	☒	A description of any assumptions or corrections, such as tests of normality and adjustment for multiple comparisons
☐	☒	A full description of the statistical parameters including central tendency (e.g. means) or other basic estimates (e.g. regression coefficient) AND variation (e.g. standard deviation) or associated estimates of uncertainty (e.g. confidence intervals)
☐	☒	For null hypothesis testing, the test statistic (e.g. *F*, *t*, *r*) with confidence intervals, effect sizes, degrees of freedom and *P* value noted<br>*Give P values as exact values whenever suitable.*
☒	☐	For Bayesian analysis, information on the choice of priors and Markov chain Monte Carlo settings
☒	☐	For hierarchical and complex designs, identification of the appropriate level for tests and full reporting of outcomes
☒	☐	Estimates of effect sizes (e.g. Cohen's *d*, Pearson's *r*), indicating how they were calculated

Our web collection on statistics for biologists contains articles on many of the points above.

Software and code

Policy information about availability of computer code

Data collection	-Confocal images were collected using Zen 3.1 (blue edition) software.
Data analysis	- Image analysis was done using ImageJ 1.53n https://imagej.nih.gov/ij/<br>-Statistic analysis was performed by Prism 7 for Windows https://www.graphpad.com/scientific-software/prism/<br>-The clean reads were mapped to the Drosophila genome sequence using Hisat2 with default parameters.<br>-The number of mapped reads were counted by featureCounts<br>- Differential gene expression analysis was performed using the DESeq2 package https://bioconductor.org/<br>-Gene ontology and KEGG pathway enrichment analyses for the differentially expressed genes were conducted using the Database DAVID https://david.ncifcrf.gov/ and PANTHER http://www.pantherdb.org/<br>-Motif analysis was done with Homer 4.11 https://homer.ucsd.edu/homer/<br>-Peaks were called using macs2 callpeak 64 and plotted using pyGenomeTracks 3.5.1 https://pygenometracks.readthedocs.io/<br>-Package ChIPseeker 1.22.1 was used to analyze the ChIP-seq data<br>-All custom scripts are available at https://github.com/YueLi9104/Metabolic-control-of-tracheal-progenitors<br>-The DOI for the Github repository is https://doi.org/10.5281/zenodo.6474350

For manuscripts utilizing custom algorithms or software that are central to the research but not yet described in published literature, software must be made available to editors and reviewers. We strongly encourage code deposition in a community repository (e.g. GitHub). See the Nature Portfolio guidelines for submitting code & software for further information.

Data

Policy information about availability of data

All manuscripts must include a data availability statement. This statement should provide the following information, where applicable:

- Accession codes, unique identifiers, or web links for publicly available datasets
- A description of any restrictions on data availability
- For clinical datasets or third party data, please ensure that the statement adheres to our policy

The authors declare that all data supporting present study, including its supplementary information files, and the source data file, are available within this article and upon reasonable request from the corresponding author. The RNA sequencing data and ChIP-seq data generated and analyzed in this study are available at NCBI at the following link: https://www.ncbi.nlm.nih.gov/geo/query/acc.cgi?acc=GSE184856. The source data underlying Figures and Supplementary Figures are provided as a Source Data file.
The databases/datasets used in the study: Drosophila genome (dm6) https://www.ncbi.nlm.nih.gov/assembly/GCF_000001215.4/

Field-specific reporting

Please select the one below that is the best fit for your research. If you are not sure, read the appropriate sections before making your selection.

☒ Life sciences ☐ Behavioural & social sciences ☐ Ecological, evolutionary & environmental sciences

For a reference copy of the document with all sections, see nature.com/documents/nr-reporting-summary-flat.pdf

Life sciences study design

All studies must disclose on these points even when the disclosure is negative.

Sample size	-In order to obtain convincing conclusions, 5-20 white pupae were used for migration experiment, EdU cell proliferation assay, and most immunofluorescence staining. Based on our preliminary experiments, 5-20 animals are sufficient for the above mentioned experiments.<br>-For glucose or ATP measurement, more than 60 larvae or pupae were detected. The sample size was determined by our preliminary experiment.<br>-To minimize the sample variation between different individuals, clusters of tracheal progenitors were collected from two L3 or pupae that were at the same developmental stage. A single cluster of tracheal progenitors is able to generate sufficient product for SMART-seq.<br>-The size number of Immunoprecipitation and ChIP-seq is 500, which grants sufficient yield for next generation sequencing. Our preliminary results revealed that the sample size needs to be larger than 150 in order to obtain enough protein/DNA for the experiments. We used 500 animals to perform three biological replicates.
Data exclusions	ChIP-seq data: one replicate (GSM5598292 : sample rep3) was excluded due to ultra low DNA concentration and poor sequencing data.<br>No other data were excluded from the analyses.
Replication	All experiments were replicated or performed independently for at least three times. Except for ChIP-seq experiments (one biological replication was not successful), other experiments were replicated independently and successfully. All the replicates of same experiment were performed independently and consecutively within a month.
Randomization	Animals were allocated to each experimental group randomly for all experiments. For experiments other than those mentioned in reporting summary, samples were randomly allocated into experimental groups.
Blinding	Image qualifications and biochemical assays were performed blindly. Blinding was not applicable to sample collection for histochemistry, migration and EdU assay, as genotypes of flies need to be visually distinguished. The investigators were blinded to group allocation during data analysis for all the experiments.

Reporting for specific materials, systems and methods

We require information from authors about some types of materials, experimental systems and methods used in many studies. Here, indicate whether each material, system or method listed is relevant to your study. If you are not sure if a list item applies to your research, read the appropriate section before selecting a response.

Materials & experimental systems

n/a	Involved in the study
☐	☒ Antibodies
☐	☒ Eukaryotic cell lines
☒	☐ Palaeontology and archaeology
☐	☒ Animals and other organisms
☒	☐ Human research participants
☒	☐ Clinical data
☒	☐ Dual use research of concern

Methods

n/a	Involved in the study
☐	☒ ChIP-seq
☒	☐ Flow cytometry
☒	☐ MRI-based neuroimaging

Antibodies

Antibodies used

-anti-β-galactosidase (mouse) (1:100) (Developmental Studies Hybridoma Bank, 40-1a)
-anti-Hnt (mouse) (1:100) (Developmental Studies Hybridoma Bank, 1G9)
-anti-Dlp (mouse) (1:100) (Developmental Studies Hybridoma Bank, 13G8)
-anti-Ncad (mouse) (1:100) (Developmental Studies Hybridoma Bank, DN-EX)
-anti-Rho1 (mouse) (1:100) (Developmental Studies Hybridoma Bank, P1D9)
-anti-MMP1 (mouse) (1:100) (Developmental Studies Hybridoma Bank, 5H7B11/3A6B4/3B8D12)
-anti-Serp (rabbit) (1:200) (Luschnig, S., 2006) from Mark Krasnow at Stanford
-anti-Verm (rabbit) (1:200) (Luschnig, S., 2006) from Mark Krasnow at Stanford
-Phospho-(Ser/Thr) Kinase Substrate Antibody Sampler Kit (rabbit) (1:1000) (Cell Signaling Technology, 9920)
-anti-FLAG (mouse) (1:1000) (Sigma, M2, F1804)
-anti-HA (mouse) (1:500) (ABclonal, AE008)
-anti-Yki (rabbit) (1:100) (Li, S., 2015) from Jin Jiang at UT Southwestern
-anti-Bnl antiserum (rabbit) (1:20 in M3) (Sutherland et al., 1996, Jarecki et al., 1999) from Mark Krasnow at Stanford
-anti-Thiophosphate ester antibody (1:5000) (Abcam, ab92570)
-anti-mouse Alexa Fluor®488 (1:200) (Jackson ImmunoResearch, 115-545-003)
-anti-Rabbit Alexa Fluor®488 (1:200) (Jackson ImmunoResearch, 111-545-003)
-anti-mouse Cyanine Cy™3 (1:200) (Jackson ImmunoResearch, 115-165-003)
-anti-Rabbit Cyanine Cy™3 (1:200) (Jackson ImmunoResearch, 111-165-003)
-anti-mouse Alexa Fluor®647 (1:200) (Jackson ImmunoResearch, 115-605-003)
-anti-Rabbit Alexa Fluor®647 (1:200) (Jackson ImmunoResearch, 111-605-003)
-HRP-conjugated α-mouse (1:5000) (Jackson ImmunoResearch, 115-035-003)
-HRP-conjugated α-rabbit (1:5000) (Jackson ImmunoResearch, 111-035-144)

Validation

All antibodies used in the study have been validated by the manufacturers or original authors:
-anti-β-galactosidase is validated by Developmental Studies Hybridoma Bank
-anti-Hnt is validated by Developmental Studies Hybridoma Bank
-anti-Dlp is validated by Developmental Studies Hybridoma Bank
-anti-Ncad is validated by Developmental Studies Hybridoma Bank
-anti-Rho1 is validated by Developmental Studies Hybridoma Bank
-anti-MMP1 is validated by Developmental Studies Hybridoma Bank
-anti-Serp (Luschnig, S., 2006) from Mark Krasnow, not commercially available
-anti-Verm (Luschnig, S., 2006) from Mark Krasnow, not commercially available
-Phospho-(Ser/Thr) Kinase Substrate Antibody Sampler Kit is validated by Cell Signaling Technology
-anti-FLAG is validated by Sigma
-anti-HA is validated byABclonal
-anti-Yki (Li, S., 2015) from Jin Jiang, not commercially available
-anti-Bnl antiserum (Sutherland et al., 1996, Jarecki et al., 1999) from Mark Krasnow, not commercially available
-anti-Thiophosphate ester antibody is validated by Abcam
-anti-mouse Alexa Fluor®488 is validated by Jackson ImmunoResearch
-anti-rabbit Alexa Fluor®488 is validated by Jackson ImmunoResearch
-anti-mouse Cyanine Cy™3 is validated by Jackson ImmunoResearch
-anti-rabbit Cyanine Cy™3 is validated by Jackson ImmunoResearch
-anti-mouse Alexa Fluor®647 is validated by Jackson ImmunoResearch
-anti-rabbit Alexa Fluor®647 is validated by Jackson ImmunoResearch
-HRP-conjugated α-mouse is validated by Jackson ImmunoResearch
-HRP-conjugated α-rabbit is validated by Jackson ImmunoResearch

Eukaryotic cell lines

Policy information about cell lines

Cell line source(s)

Commercial cell lines HEK293T from ATCC

Authentication

HEK293T cell line was bought from ATCC but were not authenticated.

Mycoplasma contamination

HEK293T cell line was tested negative for mycoplasma contamination.

March 2021

Commonly misidentified lines (See ICLAC register)	No commonly misidentified cell lines used in the study.

Animals and other organisms

Policy information about studies involving animals; ARRIVE guidelines recommended for reporting animal research

Laboratory animals	The laboratory animal involved in the study is Drosophila melanogaster. Both male and female animals were used for all experiments.<br>L3: D.melanogaster. btl-RFP-moe, btl-Gal4, Dl-Gal4, UAS-CD8:GFP, UAS-ykiS168A:HA-GFP, ex-lacZ, UAS-InRDN, UAS-Flag-yki, UAS-ykiS168A:HA-GFP, UAS-YAP-SPARK, UAS-AktRNAi, UAS-InRDN, UAS-AMPKDN<br>APF0hr: D.melanogaster. btl-RFP-moe, btl-Gal4, ex-lacZ, UAS-ykiRNAi, UAS-InRDN, UAS-Akt, dally:YFP, UAS-sdRNAi, UAS-hntRNAi, UAS-dallyRNAi, UAS-NcadRNAi, UAS-TrlRNAi, UAS-YAP-SPARK, UAS-yki.S168A<br>APF1hr: D.melanogaster. btl-RFP-moe, btl-Gal4, yki-V5-Flag<br>APF150min: D.melanogaster. btl-RFP-moe, btl-Gal4
Wild animals	No wild animals were used in the study.
Field-collected samples	No field-collected samples were used in the study.
Ethics oversight	No ethics approval is needed for experiments using Drosophila.

Note that full information on the approval of the study protocol must also be provided in the manuscript.

ChIP-seq

Data deposition

☒ Confirm that both raw and final processed data have been deposited in a public database such as GEO.

☒ Confirm that you have deposited or provided access to graph files (e.g. BED files) for the called peaks.

Data access links *May remain private before publication.*	GSE184856:<br>https://www.ncbi.nlm.nih.gov/geo/query/acc.cgi?acc=GSE184856
Files in database submission	Raw files:<br>GSM5598289 : input<br>GSM5598290 : sample rep1<br>GSM5598291 : sample rep2<br>GSM5598292 : sample rep3<br><br>Processed data:<br>Supplementary file: GSE184856_RAW.tar
Genome browser session (e.g. UCSC)	http://genome.ucsc.edu/cgi-bin/hgTracks?db=dm6&lastVirtModeType=default&lastVirtModeExtraState=&virtModeType=default&virtMode=0&nonVirtPosition=&position=chr3L%3A14582181%2D14845713&hgsid=1173218651_UBlZcOuwSwYyrLbBGy3V9BMhDTR7

Methodology

Replicates	Three experimental replicates were performed. However, GSM5598292 : sample rep3 was excluded due to low DNA concentration and poor sequencing data.
Sequencing depth	GSM5598289 : input: 61848639bp total reads, 96.52% uniquely mapped reads, paired-end<br>GSM5598290 : sample rep1: 72104093bp total reads, 95.8% uniquely mapped reads, paired-end<br>GSM5598291 : sample rep2: 67527131bp total reads, 94.9% uniquely mapped reads, paired-end<br>GSM5598292 : sample rep3: 34767184bp total reads, 82.45% uniquely mapped reads, paired-end
Antibodies	The antibody used for ChIP-seq experiment is anti-Yki from Dr. Jin Jiang laboratory (Li, S., 2015).
Peak calling parameters	Peak calling was performed using MACS2 with parameters: macs2 callpeak -f BEDPE -g dm
Data quality	GSM5598290 : sample rep1: 10597 peaks are at FDR 5%<br>GSM5598291 : sample rep2: 5620 peaks are at FDR 5%<br>GSM5598292 : sample rep3: 2260 peaks are at FDR 5%
Software	Homer (https://homer.ucsd.edu/homer/), pyGenomeTracks (https://pygenometracks.readthedocs.io/), PANTHER (http://pantherdb.org/) and package ChIPseeker were used to analyze the ChIP-seq data
