# Supplementary figures and images for: Metabolic control of progenitor cell propagation during Drosophila tracheal remodeling
Source: Nat Commun. 2022 May 20;13:2817. doi: 10.1038/s41467-022-30492-4 (PMC9122933; doi:10.1038/s41467-022-30492-4)

Fig. 6b

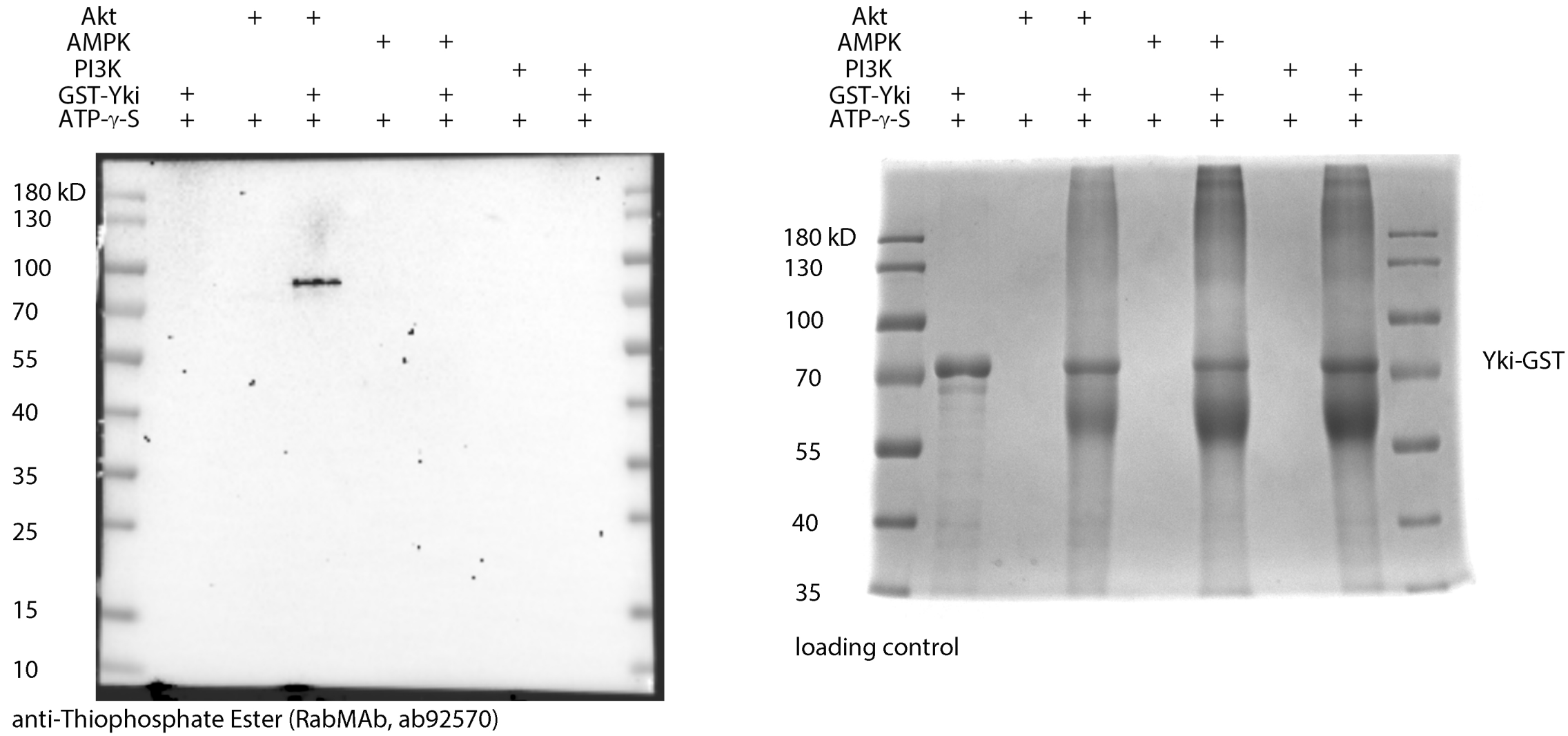

Fig. 6c

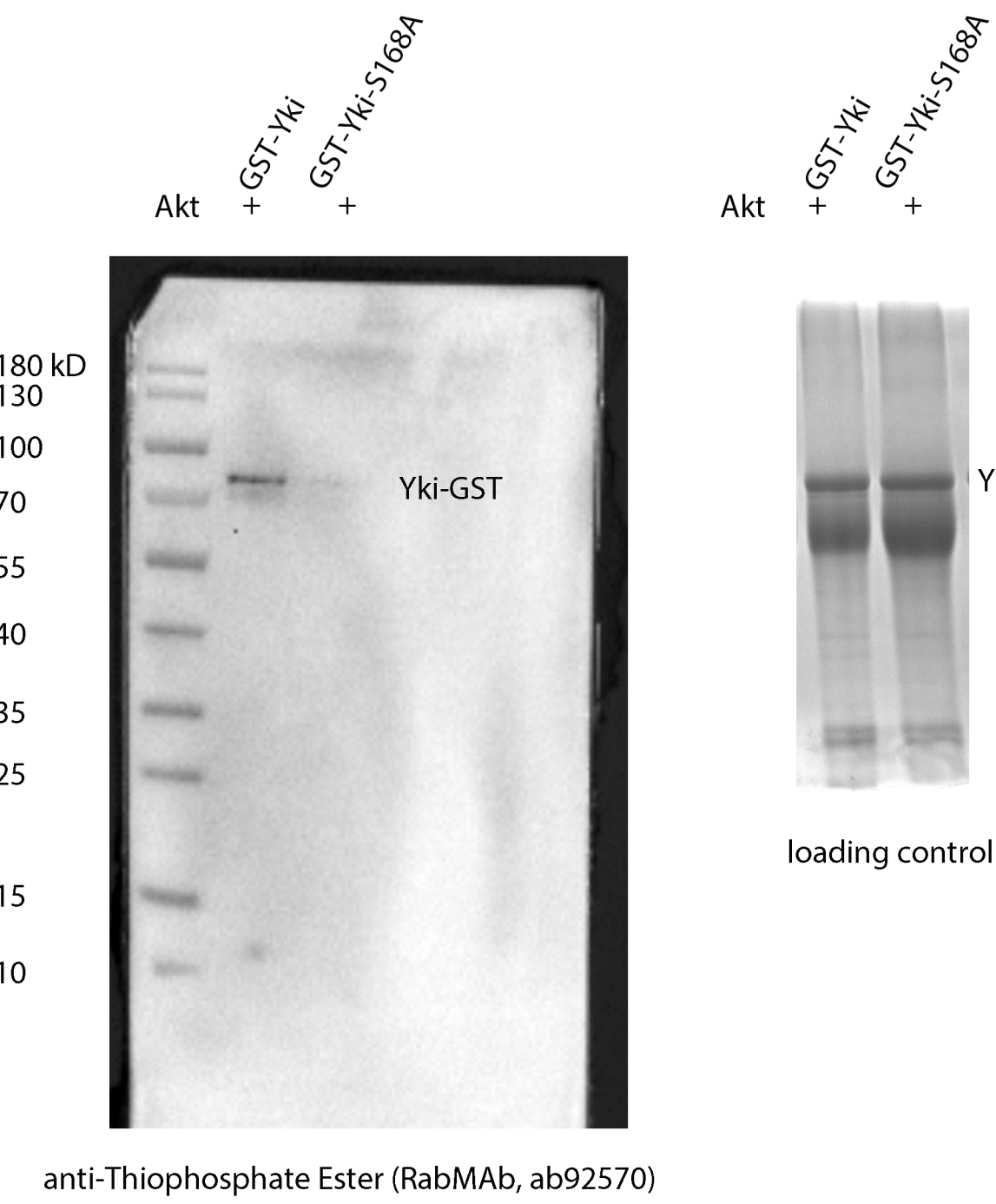

Fig. 6d

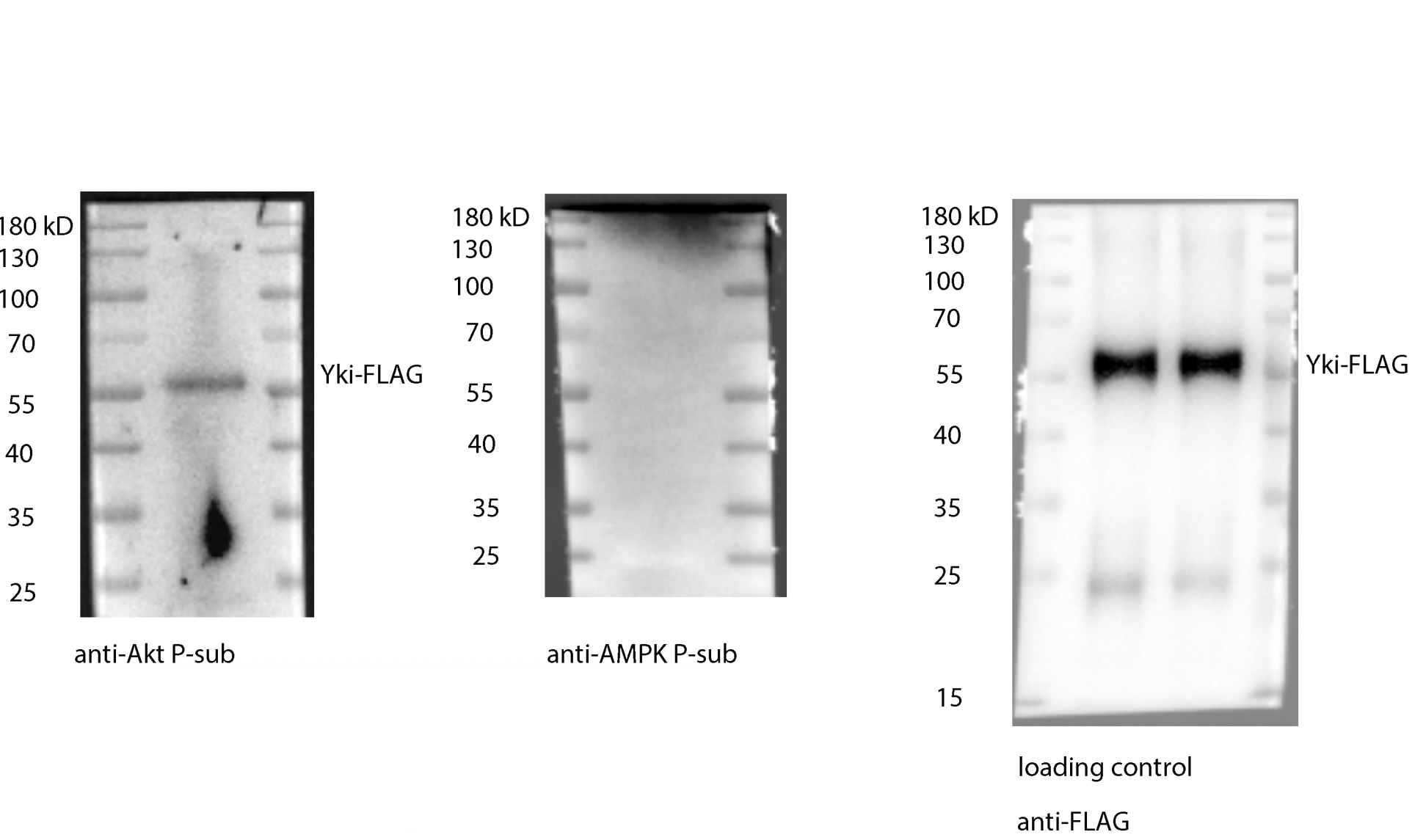

Fig. 6e

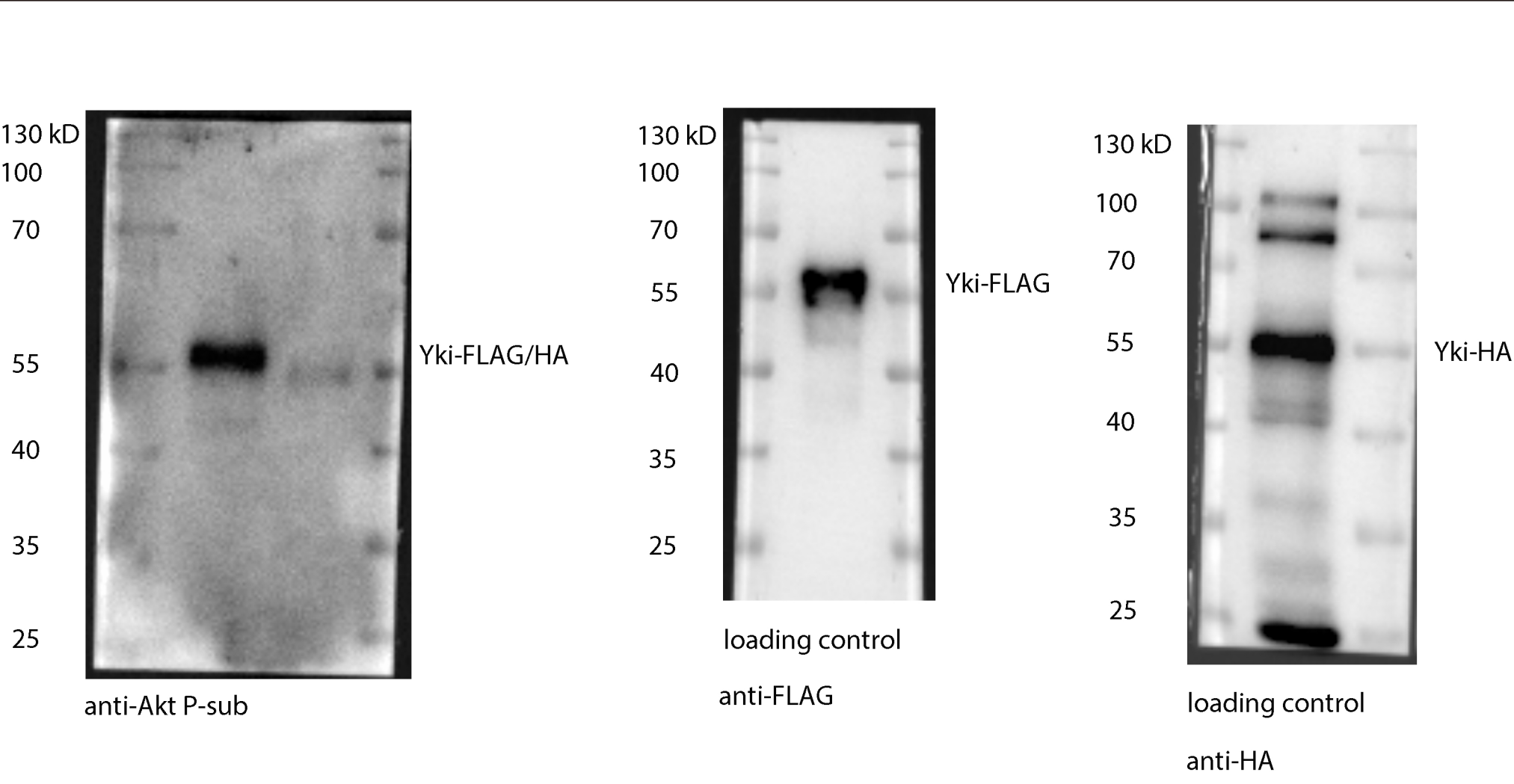

Supplement: Supplementary file 9 — Source Data [file 41467_2022_30492_MOESM9_ESM.zip › uncropped blots.pdf]
